# The Role of Astroglia in the Antidepressant Action of Deep Brain Stimulation

**DOI:** 10.3389/fncel.2015.00509

**Published:** 2016-01-12

**Authors:** Adeline Etiévant, Guillaume Lucas, Ouria Dkhissi-Benyahya, Nasser Haddjeri

**Affiliations:** ^1^Integrative and Clinical Neurosciences EA481, University of Bourgogne Franche-ComtéBesançon, France; ^2^CHRU BesançonBesançon, France; ^3^Institut François Magendie, Institut National de la Santé et de la Recherche Médicale U862, University of BordeauxBordeaux, France; ^4^Stem Cell and Brain Research Institute, Institut National de la Santé et de la Recherche Médicale U1208Bron, France; ^5^University of Lyon, University of Lyon ILyon, France

**Keywords:** antidepressant, DBS mechanism of action, astrocytes, gliotransmission, adenosine

With more than 350 million depressed individuals worldwide, major depressive disorder is one of the most common psychiatric illnesses. Although, the pathophysiology of depression is far from being fully understood, five decades of development of different classes of antidepressants targeting central monoaminergic systems (serotonin, noradrenaline and dopamine) has led to the emergence of the monoaminergic hypothesis. However, despite a growing number of available pharmacotherapies, treatment of major depression nevertheless remains unsatisfactory.

## Role of glia in the physiopathology of depression and the mechanisms of action of antidepressants

Ten years ago it was postulated that abnormal functioning of glial cells, particularly of astrocytes, contribute to the physiopathology of depression (for review, Rajkowska and Miguel-Hidalgo, 2007). Structural and functional abnormalities of glial cells were found in brains of post-mortem depressed patients and in animal models of depression (Czéh et al., 2006, 2007; Banasr et al., 2007; Sun et al., 2012). Moreover, it has been shown that a loss or a functional alteration of astrocytes in the prefrontal cortex is sufficient to induce depressive-like behaviors in rodents (Banasr and Duman, 2008; Sun et al., 2012; Kong et al., 2014).

Given the intimate anatomical and functional relationships between astrocytes and neurons, a tempting hypothesis has emerged proposing that the effects of antidepressant therapies can be, at least in part, mediated by direct astrocytic modulations of neuronal networks. In support of this idea, increasing experimental evidence suggests that antidepressants induce functional changes in astrocytes (Czéh and Di Benedetto, 2012). In addition, it is becoming increasingly clear that the astrocytic network is able to regulate neuronal activity and synaptic transmission through the release of gliotransmitters at what is now called “the tripartite synapse” (Halassa and Haydon, 2010; Panatier et al., 2011). Astrocytes can be directly modulated by an antidepressant treatment or indirectly activated by antidepressant-induced increases in neurotransmitter concentrations in the synaptic cleft, leading to the activation of G protein–coupled receptors (including serotonergic, adrenergic and dopaminergic receptors) on astrocyte membranes. Once “activated,” astrocytes release gliotransmitters and modulate neuronal communication and antidepressant responses. The concept of gliotransmission precisely refers to the process by which astrocytes release chemical factors in the vicinity of synapses thus modulating the activity of neighboring cells (for review, Volterra and Meldolesi, 2005; Santello et al., 2012). Although, a large number of such molecules have already been identified (e.g., glutamate, ATP, D-serine, GABA, neurotrophins), our focus here will be specifically on adenosine since it acts directly on the process of neuronal communication and is implicated in pseudo-depressive-like behavior and antidepressant response.

ATP is mainly considered as an excitatory transmitter (Gordon et al., 2005) but it is also rapidly hydrolyzed into adenosine by ectonucleotidases present in the synaptic cleft. Adenosine acts as a powerful inhibitor of excitatory transmission through the stimulation of adenosine A_1_ receptors (Newman, 2003; Pascual et al., 2005). Adenosine or adenosine agonists induce depressive-like behaviors in two experimental paradigms, namely inescapable shocks (Minor et al., 1994; Hunter et al., 2003) and forced swimming tests (Kulkarni and Mehta, 1985; Cao et al., 2013), an effect that can be prevented by specific adenosine antagonists or antidepressants. Specific A_2A_ receptor antagonists also reverse synaptic changes induced by stress in the hippocampus, which is considered as a preclinical marker of antidepressant responses (Cunha et al., 2006). However, these interpretations are further complicated by the findings observed after selective manipulations of the adenosine A_1_ transmission. Thus, central administration of an A_1_ receptor agonist mimics the antidepressant effect of sleep deprivation, an effect absent in ${\text{A}}_{1}^{-∕-}$ KO mice (Hines et al., 2013). Sleep deprivation is also associated with pronounced increases of adenosine levels and an up-regulation of glial adenosine A_1_-receptors in the brains of both depressed patients (Van Calker and Biber, 2005) and rodents (Hines et al., 2013). The apparent discrepancy between the “depressiogenic” influence of adenosine, and some antidepressant-like actions of A_1_ agonists, could be due to the complex modulation exerted by A_1_ receptors on axonal firing that appears to depend on the degree of activity of the related networks (see below).

## Astrocytes are deeply involved in the neurobiological effects of deep brain stimulation

As observed during pharmacological treatments, recent data shows that astrocyte function can be modulated by deep brain stimulation (DBS), a non-pharmacological antidepressant intervention. DBS is an invasive brain stimulation technique considered as a new hope in the treatment of several intractable psychiatric diseases such as major depression (Mayberg et al., 2005; Puigdemont et al., 2015). Current research is mainly focused on the effects of DBS on neurons, i.e., how myelinated and unmyelinated axons, dendrites and neuronal cell bodies respond to DBS (Mcintyre et al., 2004; Gubellini et al., 2009). However, the role of astrocytes in this context has not yet been addressed. Several arguments support the view that the effects of DBS can, at least in part, be mediated by astrocytes acting on neuronal networks (for review, Vedam-Mai et al., 2012). First, it is well known that DBS modulates regional blood flow in the stimulated area, an effect that can be considered as a direct manifestation of changes in astrocytic activity (Kefalopoulou et al., 2010). Second, astrocytes can be directly activated by high frequency stimulation, leading to a rapid Ca^2+^ increase (Kang et al., 1998; Serrano et al., 2006, 2008). Third, high frequency stimulation of primary astrocytes *in vitro* results in calcium waves and release of glutamate and ATP (Tawfik et al., 2010). Accordingly, Bekar et al. (2008) have shown *in vitro* that DBS was associated with an increase of ATP outflow within the thalamus, resulting in an accumulation of adenosine, which in turn depressed excitatory transmission through A_1_ receptors activation. The authors proposed that, once present in the synaptic cleft, adenosine would activate post-synaptic A_1_ receptors positively coupled to K^+^ channels and pre-synaptic A_1_ receptors negatively associated with Ca^2+^ channels. Both actions would result in the inhibition of neuronal communication (Pascual et al., 2005).

Our recent investigations suggest that astrocytes are deeply involved in the antidepressant-like effects of DBS in rats. The antidepressant response induced by DBS in humans can be modeled in rats by stimulating the infralimbic part of the prefrontal cortex (IL-PFC). It has been shown that acute DBS produced an antidepressant-like effect in the forced swim test (Etiévant et al., 2015) and that chronic DBS is able to reverse the depressive-like states observed in Flinders sensitive Line rats (Rea et al., 2014) or induced by chronic mild stress (Hamani et al., 2012). Therefore, the antidepressant-like effect of DBS is associated with the occurrence of *in vivo* pre-clinical markers (Etiévant et al., 2015). We showed that acute DBS induced a rapid increase of hippocampal neurogenesis, reversed the effects of stress on hippocampal synaptic metaplasticity, increased spontaneous IL-PFC low-frequency oscillations and both raphe 5-HT firing activity and synaptogenesis. Significantly, we demonstrated that DBS-induced neural adaptations are strongly altered by pharmacological ablation of astrocytes within the site of stimulation (IL-PFC). Glial lesion with the gliotoxin L-alpha amino-adipic acid (L-AAA) counteracted the behavioral effect of high frequency DBS in the forced swim test and all above cited markers of the antidepressant response. We also found that DBS-induced antidepressant-like response was prevented by IL-PFC neuronal lesion and gap junction blockade as well as by adenosine A_1_ receptor antagonists including caffeine.

**Figure 1:**
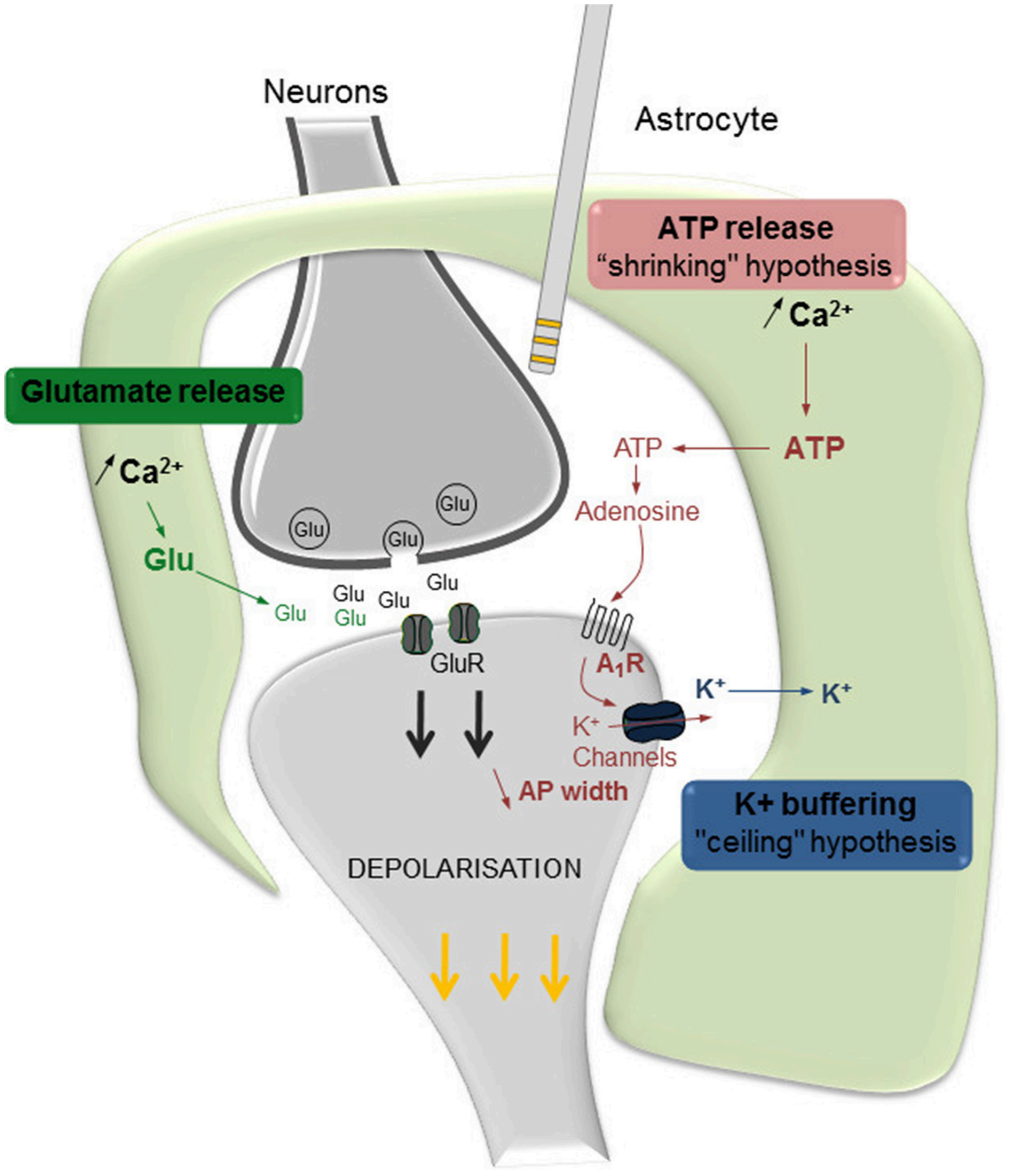
**Proposed hypotheses regarding the involvement of astrocytes in the effects of DBS**. Once activated by DBS, astrocytes communicate with neurons at the synapse level and regulate the effects of DBS. Astrocytes, by releasing glutamate (Glu), stimulate neuronal synaptic release and contribute to the activation of post-synaptic receptors (in green). ATP is rapidly hydrolyzed into adenosine, which increases the stimulation of adenosine A_1_ receptors (A_1_R) and, in turn, results in a K^+^ channel-mediated reduction of the late hyperpolarization phase of action potentials (in red). Ultimately, the resulting temporal shrinkage of action potentials (AP width) may help the neuron to sustain the high frequency demand related to IL-DBS. Astrocytes also maintain K^+^ homeostasis, by actively pumping K^+^ ions from the extracellular level thus preventing their accumulation due to neuronal activity (in blue). Modified from Etiévant et al. (2015).

An elegant review discussing the role of astrocytes in the effects of DBS (Vedam-Mai et al., 2012) recently raised the hypothesis that astrocytes, once activated by electrical stimulation, would release ATP and glutamate leading to an inhibition or an excitation of synaptic transmission, respectively. Recent data partially supports this hypothesis and offers further insights. Our *in vivo* electrophysiological results revealed that the astroglial modulation of DBS involved mechanisms related to changes in adenosine A_1_ receptor function, together with the elevation of extracellular K^+^ concentration (Etiévant et al., 2015). Our results further showed that the enhancing effect of bilateral DBS on 5-HT neuronal activity was potentiated by a selective A_1_ receptor agonist, unilaterally infused during the stimulation. This result, together with the fact that the A_1_ receptor antagonist DPCPX prevents the antidepressant-like effect of DBS in the forced swim test, indicates that the efficacy of DBS partially depends on adenosine A_1_ receptor stimulation. Interestingly, recent studies aimed to characterize the role played by A_1_ receptors in the shape of action potentials and the regulation of axonal conductance report that the administration of an adenosine antagonist increases the width of axonal action potentials. This result suggests that astrocytes, through the release of adenosine and subsequent A_1_ receptor stimulation, are able to modulate the shape of axonal action potentials, shortening the total duration of the spike and “shrinking” its shape (Sasaki et al., 2011). This latter effect could be due to a modulation of the voltage-activated K^+^ channels responsible for neuronal after hyperpolarization. It has been proposed that such a “temporal shrinking” of action potentials can be beneficial when the neuron is solicited in response to high-frequency stimulations, allowing to sustain bursting activity that requires very short inter-spike intervals (Sasaki et al., 2011). Thus, we have proposed that a loss of astrocytes within the site of stimulation induces a drop of adenosine extracellular concentrations and an altered temporal shrinking of action potentials responsible for the alteration of the neurobiological effects of DBS (Figure 1).

Since astrocytes are able to maintain the potassium homeostasis by actively pumping K^+^ ions from the extracellular space (Kofuji and Newman, 2004), we hypothesized that an alteration of astrocyte function within the lesioned site leads to an accumulation of extracellular K^+^ which, in turn, would produce a depolarization of neuron membrane and a blockade of DBS-mediated effects. Hence in our *in vivo* study, a K^+^-enriched aCSF was perfused within the IL-PFC using reverse dialysis while recording 5-HT neurons. The obtained results confirmed our hypothesis of a “ceiling effect,” related to a K^+^-dependent depolarization of pyramidal neurons, since high frequency DBS is unable to further affect 5-HT activity in the presence of high [K^+^]. Thus, the depolarizing action of an elevated extracellular [K^+^] potentially impairs the ability of pyramidal cells to respond to the phasic, high-frequency solicitation demands of sustained electrical stimulations of 130 Hz. This effect is frequency-dependent since both the 5-HT-activating and the behavioral effectiveness in the forced swim test of a 30 Hz DBS remained unaltered in glial-lesioned rats and in high [K^+^] conditions. This result strongly suggests that, in the absence of astrocytes, the depolarization of neuronal membrane related to K^+^ accumulation fails to reach a supra-threshold, “depolarization block-like” level, and that pyramidal neurons are still able to follow a low frequency DBS (30 Hz; Etiévant et al., 2015).

## Conclusion

The astroglial system plays a crucial role in the mechanisms of action of DBS. Accordingly, the antidepressant-like response induced by DBS is counteracted by a pharmacological lesion of astrocytes in the stimulated area. Two mechanistic hypotheses have been proposed to explain the astrocytic modulation of the neuronal response induced by DBS (Figure 1). First, the “shrinking hypothesis” suggests that astrocytes, by releasing adenosine in response to DBS, activate neuronal A_1_ receptors resulting in a shortening of the width of action potentials. Second, the “ceiling hypothesis” proposes that astrocytes, by actively pumping K^+^ ions from the extracellular spaces, prevent the establishment of the “depolarization-like blockade” of the neuronal membrane. Both events are directed to an optimal functioning of pyramidal neurons that are still capable of following high frequency stimulations induced by DBS. Lastly as a translational outcome, we have proposed that an unaltered neuronal–glial system constitutes a major prerequisite to optimize antidepressant DBS efficacy, and that decreasing the frequency of DBS would increase the antidepressant response of partial responders.

## Author contributions

All the authors participated to the conception and the content of the opinion. AE wrote the opinion with the help of GL, OD, and NH. All the authors revised critically the manuscript and gave their approval for publication.

### Conflict of interest statement

The authors declare that the research was conducted in the absence of any commercial or financial relationships that could be construed as a potential conflict of interest.

## References

[B1] Banasr M. Duman R. S. (2008). Glial loss in the prefrontal cortex is sufficient to induce depressive-like behaviors. Biol. Psychiatry 64, 863–870. 10.1016/j.biopsych.2008.06.008 18639237 PMC2709733

[B2] Banasr M. Valentine G. W. Li X. Y. Gourley S. L. Taylor J. R. Duman R. S. (2007). Chronic unpredictable stress decreases cell proliferation in the cerebral cortex of the adult rat. Biol. Psychiatry 62, 496–504. 10.1016/j.biopsych.2007.02.006 17585885

[B3] Bekar L. Libionka W. Tian G. F. Xu Q. Torres A. Wang X.. (2008). Adenosine is crucial for deep brain stimulation-mediated attenuation of tremor. Nat. Med. 14, 75–80. 10.1038/nm1693 18157140

[B4] Cao X. Li L. P. Wang Q. Wu Q. Hu H. H. Zhang M.. (2013). Astrocyte-derived ATP modulates depressive-like behaviors. Nat. Med. 19, 773–777. 10.1038/nm.3162 23644515

[B5] Cunha G. M. Canas P. M. Oliveira C. R. Cunha R. A. (2006). Increased density and synapto-protective effect of adenosine A2A receptors upon sub-chronic restraint stress. Neuroscience 141, 1775–1781. 10.1016/j.neuroscience.2006.05.024 16797134

[B6] Czéh B. Di Benedetto B. (2012). Antidepressants act directly on astrocytes: evidences and functional consequences. Eur. Neuropsychopharmacol. 23, 171–185. 10.1016/j.euroneuro.2012.04.017 22609317

[B7] Czéh B. Müller-Keuker J. I. Rygula R. Abumaria N. Hiemke C. Domenici E.. (2007). Chronic social stress inhibits cell proliferation in the adult medial prefrontal cortex: hemispheric asymmetry and reversal by fluoxetine treatment. Neuropsychopharmacology 32, 1490–1503. 10.1038/sj.npp.1301275 17164819

[B8] Czéh B. Simon M. Schmelting B. Hiemke C. Fuchs E. (2006). Astroglial plasticity in the hippocampus is affected by chronic psychosocial stress and concomitant fluoxetine treatment. Neuropsychopharmacology 31, 1616–1626. 10.1038/sj.npp.1300982 16395301

[B9] Etiévant A. Oosterhof C. A. Bétry C. Abrial E. Novo-Perez M. Rovera R.. (2015). Astroglial control of the antidepressant-like effects of prefrontal cortex deep brain stimulation. EBiomedicine 2, 896–906. 10.1016/j.ebiom.2015.06.023 26425697 PMC4563138

[B10] Gordon G. R. Baimoukhametova D. V. Hewitt S. A. Rajapaksha W. R. Fisher T. E. Bains J. S. (2005). Norepinephrine triggers release of glial ATP to increase postsynaptic efficacy. Nat. Neurosci. 8, 1078–1086. 10.1038/nn1498 15995701

[B11] Gubellini P. Salin P. Kerkerian-Le Goff L. Baunez C. (2009). Deep brain stimulation in neurological diseases and experimental models: from molecule to complex behavior. Prog. Neurobiol. 89, 79–123. 10.1016/j.pneurobio.2009.06.003 19559747

[B12] Halassa M. M. Haydon P. G. (2010). Integrated brain circuits: astrocytic networks modulate neuronal activity and behavior. Annu. Rev. Physiol. 72, 335–355. 10.1146/annurev-physiol-021909-135843 20148679 PMC3117429

[B13] Hamani C. Machado D. C. Hipólide D. C. Dubiela F. P. Suchecki D. Macedo C. E.. (2012). Deep brain stimulation reverses anhedonic-like behavior in a chronic model of depression: role of serotonin and brain derived neurotrophic factor. Biol. Psychiatry 71, 30–35. 10.1016/j.biopsych.2011.08.025 22000731 PMC5756076

[B14] Hines D. J. Schmitt L. I. Hines R. M. Moss S. J. Haydon P. G. (2013). Antidepressant effects of sleep deprivation require astrocyte-dependent adenosine mediated signaling. Transl. Psychiatry 3, e212. 10.1038/tp.2012.136 23321809 PMC3566717

[B15] Hunter A. M. Balleine B. W. Minor T. R. (2003). Helplessness and escape performance: glutamate-adenosine interactions in the frontal cortex. Behav. Neurosci. 117, 123–135. 10.1037/0735-7044.117.1.123 12619915

[B16] Kang J. Jiang L. Goldman S. A. Nedergaard M. (1998). Astrocyte-mediated potentiation of inhibitory synaptic transmission. Nat. Neurosci. 1, 683–692. 10.1038/3684 10196584

[B17] Kefalopoulou Z. Paschali A. Markaki E. Ellul J. Chroni E. Vassilakos P.. (2010). Regional cerebral blood flow changes induced by deep brain stimulation in secondary dystonia. Acta Neurochir. (Wien.) 152, 1007–1014. 10.1007/s00701-010-0612-y 20182892

[B18] Kofuji P. Newman E. A. (2004). Potassium buffering in the central nervous system. Neuroscience 129, 1045–1056. 10.1016/j.neuroscience.2004.06.008 15561419 PMC2322935

[B19] Kong H. Zeng X. N. Fan Y. Yuan S. T. Ge S. Xie W. P.. (2014). Aquaporin-4 knockout exacerbates corticosterone-induced depression by inhibiting astrocyte function and hippocampal neurogenesis. CNS Neurosci. Ther. 20, 391–402. 10.1111/cns.12222 24422972 PMC6493035

[B20] Kulkarni S. K. Mehta A. K. (1985). Purine nucleoside–mediated immobility in mice: reversal by antidepressants. Psychopharmacology (Berl.) 85, 460–463. 10.1007/BF00429665 2991960

[B21] Mayberg H. S. Lozano A. M. Voon V. Mcneely H. E. Seminowicz D. Hamani C.. (2005). Deep brain stimulation for treatment-resistant depression. Neuron 45, 651–660. 10.1016/j.neuron.2005.02.014 15748841

[B22] Mcintyre C. C. Savasta M. Kerkerian-Le Goff L. Vitek J. L. (2004). Uncovering the mechanism(s) of action of deep brain stimulation: activation, inhibition, or both. Clin. Neurophysiol. 115, 1239–1248. 10.1016/j.clinph.2003.12.024 15134690

[B23] Minor T. R. Winslow J. L. Chang W. C. (1994). Stress and adenosine: II. Adenosine analogs mimic the effect of inescapable shock on shuttle-escape performance in rats. Behav. Neurosci. 108, 265–276. 10.1037/0735-7044.108.2.265 8037870

[B24] Newman E. A. (2003). Glial cell inhibition of neurons by release of ATP. J. Neurosci. 23, 1659–1666. 12629170 10.1523/JNEUROSCI.23-05-01659.2003 PMC2322877

[B25] Panatier A. Vallée J. Haber M. Murai K. K. Lacaille J. C. Robitaille R. (2011). Astrocytes are endogenous regulators of Basal transmission at central synapses. Cell 146, 785–798. 10.1016/j.cell.2011.07.022 21855979

[B26] Pascual O. Casper K. B. Kubera C. Zhang J. Revilla-Sanchez R. Sul J. Y.. (2005). Astrocytic purinergic signaling coordinates synaptic networks. Science 310, 113–116. 10.1126/science.1116916 16210541

[B27] Puigdemont D. Portella M. Pérez-Egea R. Molet J. Gironell A. De Diego-Adeliño J.. (2015). A randomized double-blind crossover trial of deep brain stimulation of the subcallosal cingulate gyrus in patients with treatment-resistant depression: a pilot study of relapse prevention. J. Psychiatry Neurosci. 40, 224–231. 10.1503/jpn.130295 25652752 PMC4478055

[B28] Rajkowska G. Miguel-Hidalgo J. J. (2007). Gliogenesis and glial pathology in depression. CNS Neurol. Disord. Drug Targets 6, 219–233. 10.2174/187152707780619326 17511618 PMC2918806

[B29] Rea E. Rummel J. Schmidt T. T. Hadar R. Heinz A. Mathé A. A.. (2014). Anti-anhedonic effect of deep brain stimulation of the prefrontal cortex and the dopaminergic reward system in a genetic rat model of depression: an intracranial self-stimulation paradigm study. Brain Stimul. 7, 21–28. 10.1016/j.brs.2013.09.002 24139146

[B30] Santello M. Calí C. Bezzi P. (2012). Gliotransmission and the tripartite synapse. Adv. Exp. Med. Biol. 970, 307–331. 10.1007/978-3-7091-0932-8_14 22351062

[B31] Sasaki T. Matsuki N. Ikegaya Y. (2011). Action-potential modulation during axonal conduction. Science 331, 599–601. 10.1126/science.1197598 21292979

[B32] Serrano A. Haddjeri N. Lacaille J. C. Robitaille R. (2006). GABAergic network activation of glial cells underlies hippocampal heterosynaptic depression. J. Neurosci. 26, 5370–5382. 10.1523/JNEUROSCI.5255-05.2006 16707789 PMC6675310

[B33] Serrano A. Robitaille R. Lacaille J. C. (2008). Differential NMDA-dependent activation of glial cells in mouse hippocampus. Glia 56, 1648–1663. 10.1002/glia.20717 18618659

[B34] Sun J. D. Liu Y. Yuan Y. H. Li J. Chen N. H. (2012). Gap junction dysfunction in the prefrontal cortex induces depressive-like behaviors in rats. Neuropsychopharmacology 37, 1305–1320. 10.1038/npp.2011.319 22189291 PMC3306892

[B35] Tawfik V. L. Chang S. Y. Hitti F. L. Roberts D. W. Leiter J. C. Jovanovic S.. (2010). Deep brain stimulation results in local glutamate and adenosine release: investigation into the role of astrocytes. Neurosurgery 67, 367–375. 10.1227/01.NEU.0000371988.73620.4C 20644423 PMC2919357

[B36] Van Calker D. Biber K. (2005). The role of glial adenosine receptors in neural resilience and the neurobiology of mood disorders. Neurochem. Res. 30, 1205–1217. 10.1007/s11064-005-8792-1 16341582

[B37] Vedam-Mai V. Van Battum E. Y. Kamphuis W. Feenstra M. G. Denys D. Reynolds B. A.. (2012). Deep brain stimulation and the role of astrocytes. Mol. Psychiatry 17, 124–131. 10.1038/mp.2011.61 21625231

[B38] Volterra A. Meldolesi J. (2005). Astrocytes, from brain glue to communication elements: the revolution continues. Nat. Rev. Neurosci. 6, 626–640. 10.1038/nrn1722 16025096

